# Global Optimal Structured Embedding Learning for Remote Sensing Image Retrieval

**DOI:** 10.3390/s20010291

**Published:** 2020-01-04

**Authors:** Pingping Liu, Guixia Gou, Xue Shan, Dan Tao, Qiuzhan Zhou

**Affiliations:** 1 College of Computer Science and Technology, Jilin University, Changchun 130012, China; gougx18@mails.jlu.edu.cn (G.G.); shanxue19@mails.jlu.edu.cn (X.S.); 2 Key Laboratory of Symbolic Computation and Knowledge Engineering of Ministry of Education, Jilin University, Changchun 130012, China; 3 School of Mechanical Science and Engineering, Jilin University, Changchun 130025, China; 4 School of Electronic and Information Engineering, Beijing Jiaotong University, Beijing 100044, China; dtao@bjtu.edu.cn; 5 College of Communication Engineering, Jilin University, Changchun 130012, China; zhouqz@jlu.edu.cn

**Keywords:** remote sensing image retrieval, convolutional neural network, deep metric learning, global optimization

## Abstract

A rich line of works focus on designing elegant loss functions under the deep metric learning (DML) paradigm to learn a discriminative embedding space for remote sensing image retrieval (RSIR). Essentially, such embedding space could efficiently distinguish deep feature descriptors. So far, most existing losses used in RSIR are based on triplets, which have disadvantages of local optimization, slow convergence and insufficient use of similarity structure in a mini-batch. In this paper, we present a novel DML method named as global optimal structured loss to deal with the limitation of triplet loss. To be specific, we use a softmax function rather than a hinge function in our novel loss to realize global optimization. In addition, we present a novel optimal structured loss, which globally learn an efficient deep embedding space with mined informative sample pairs to force the positive pairs within a limitation and push the negative ones far away from a given boundary. We have conducted extensive experiments on four public remote sensing datasets and the results show that the proposed global optimal structured loss with pairs mining scheme achieves the state-of-the-art performance compared with the baselines.

## 1. Introduction

The deep development of remote sensing technology in recent years has induced urgent demands for processing, analyzing and understanding the high-resolution remote sensing images. The most fundamental and key task for remote sensing image analysis (RSIA) is to recognize, detect, classify and retrieve the images belonging to multiple remote sensing categories like agricultural, airplane, forest and so on [1,2,3,4,5]. Among all these tasks, remote sensing image retrieval (RSIR) [2,6,7,8] is the most challengeable in analyzing remote sensing data effectively. The main target of RSIR is to retrieve image through a given remote sensing dataset for a query and return the images with the similar visual information. RSIR has become more and more attractive due to the explosive increase in the volume of high-quality remote sensing images in the last decades [2,5,8].

Compared with content-based image retrieval (CBIR), RSIR is more challenging as there are vast geographic areas containing far-ranging semantic instances with subtle difference which is difficult to distinguish. Moreover, the images which belong to the same visual category might vary in positions, scales and appearances largely. The most key and urgent challenge is to extract more compact and discriminative feature representations to efficiently measure the similarity between the query image and retrieval images. There are large amounts of researches focusing on discriminative features extraction which have made tremendous progress by incorporating the effective methods used in the field of general image retrieval [5,9,10]. In the early times, researchers tended to utilize the characteristics like spectral, shape and texture to extract low-level feature representations [11,12,13,14]. However, these representations are hard to extract as the great demand for domain professional knowledge and excellent manual skills. And then, more superior mid-level features were proposed to enhance the performance of RSIR. The mid-level features are mainly based on the local descriptor of scale-invariant feature transform (SIFT) [15], which might maintain invariance with the change of translation, illumination and occlusion compared with the low-level feature. A large number of aggregation approaches were used to encode SIFT descriptors to generate mid-level features in the task of RSIR, including bag-of-words (BoW) [16,17], vector of locally aggregated descriptor (VLAD) [18] and fisher kernels (FK) [9,19]. These mid-level handcraft features always contain insufficient visual clues and these feature representations are ineffective to promote the performance of RSIR.

With the remarkable successful attempt in utilizing AlexNet for the task of general image classification [20], the convolutional neural network (CNN) has been widely adopted to extract high-level feature representations for promoting the effectiveness of general image retrieval tasks in the last decades [21,22,23,24,25]. With the development of deep learning research, it has been introduced to the tasks of RSIA like recognition [26,27], classification [1,5,28,29,30] and retrieval [2,5,31,32]. The high-level discriminative feature representations extracted from CNN with metric learning are more and more frequently used to boost the performance of RSIR [30,33,34,35,36,37]. Deep metric learning (DML) is an efficient approach which forces the images close to the similar visual information and pushes the dissimilar ones far away from each other [38,39,40]. The key challenge for DML is how to design an informative sample pairs mining strategy and an effective loss function to learn a discriminative embedding space. The pairwise loss functions is a common option used in image retrieval which constructs the training samples into pairs, such as contrastive loss [41], triplet loss [42], N-pairs loss [43], lifted structured loss [44], multi-similarity loss [45] and ranked list loss [46]. The lifted structured loss [44] targeted to utilize a smooth loss function which take the information of all sampled pairs into consideration, but this loss could hardly keep the structured distribution within the intraclasses, and the difference between positive and negative sample pairs might weaken the distinctiveness of the learned deep embedding space. Although ranked list loss [46] has made full use of structured information inside the training mini-batch, it fails to consider the relationship between positive and negative sample pairs. Recently, there are some attempts tending to utilize contrastive and triplet loss to fine-tune the network model for the task of RSIR and have obtained appreciable performance [33,34,35].

**Figure 1:**
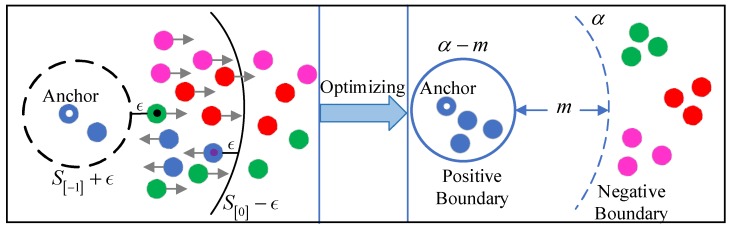
The optimization process under the proposed global optimal structured loss. The circles with different colors denote the samples with different label. The left part is the original distribution of sample pairs. The blue circle with small white circle in the center is the anchor, the green circle with small black circle in the center is the hardest negative sample to the anchor and the similarity of them is $S_{\left[-1\right]}$, the blue circle with small purple circle in the center is the hardest positive samples to the anchor and the similarity of them is $S_{\left[0\right]}$. We use pairs mining strategy to sample more informative pairs for optimization. The black solid line is the negative border for negative pairs mining and the black dot line is the positive border for positive pairs mining. The cycles with arrow denote the mined informative samples and the arrows are the gradient direction. The right part is distribution optimization. The blue solid line is positive boundary used to limit positive pairs within a hypersphere. The blue dot line is negative boundary used to pull negative pairs far away from anchor.

However, the performance of RSIR still does not fully meet the demand as there are a few limitations in these pair-based structured losses. Firstly, most of the existing pair-based losses take all samples into consideration, which might lead to slow convergence and weaken the robustness of network model [41,42,47]. To address this issue, we utilize an efficient pairs mining strategy to select more informative sample pairs to improve the performance of RSIR. Secondly, most methods construct the samples locally inside a mini-batch and fail to make full use of the information of sample pairs during training [41,42,43,47,48]. To make full use of the informative sample pairs, we exploit all samples in a training mini-batch as anchors to select informative sample pairs and utilize them to obtain a boosted performance in the task of RSIR. Thirdly, to efficiently meet the key challenge of high interclass (low intraclass) similarity exhibiting, we propose a novel global optimal structured loss to globally learn a discriminative embedding space by introducing softmax loss into RSIR. It aims at limiting the positive sample pairs into a given hypersphere and separating the negative and positive sample pairs by a certain margin. It is effective to enlarge intraclass compactness and interclass separability. Our global optimal structured loss with informative pairs mining strategy is shown in Figure 1. The proposed novel pair-based loss function takes the advantages of lifted structured loss and ranked list loss at the same time and is effective in optimizing the network model by making full use of the information of sampled pairs and maintaining the similarity structure inside a mini-batch simultaneously. Furthermore, to unify the metric during training and testing stage, we utilize inner product to measure the similarity between two remote sensing images.

As illustrated above, in our paper, we make the following contributions to improve the performance of RSIR task:

(1) We propose to use a softmax function in our novel loss to solve the key challenge of local optimum in most methods. This is efficient to realize global optimization which could be significant to enhance the performance of RSIR.
(2) We present a novel optimal structured loss to globally learn an efficient deep embedding space with mined informative sample pairs to force the positive pairs within a limitation and push the negative ones far away from a given boundary. During training stage, we take the information of all these selected sample pairs and the difference between positive and negative pairs into consideration; make the intraclass samples more compact and the interclass ones more separated while preserving the similarity structure of samples.
(3) To further reveal the effectiveness of the RSIR task under DML paradigm, we perform the task of RSIR with various commonly used metric loss functions on the public remote sensing datasets. These loss functions aim at fine-tuning the pre-trained network to be more adaptive for a certain task. The results show that the proposed method achieves outstanding performance which would be reported in experiments section.
(4) To verify the superiority of our proposed optimal structured loss, we conduct the experiment on multiple remote sensing datasets. The retrieval performance is boosted with approximately 5% on these public remote sensing datasets compared with the existing methods [28,49,50,51] and this demonstrates that our proposed method achieves the state-of-the-art results in the task of RSIR.

We would like to present the organization of our paper as follows: We describe the related work from the aspects of metric learning and methods used in RSIR in Section 2. We give a detailed interpretation of our proposed method and the framework of the RSIR with our method in Section 3. In Section 4, we give some details of our experiments and present their results and analysis. Lastly, we present the conclusions of our paper.

## 2. Related Work

In this section, we make a summary of various works related to DML and the task of RSIR. Firstly, we introduce some work about clustering-based losses, pair-based structured losses and informative pairs mining strategies. Then, we provide an overview on the development of RSIR which is based on handcraft and deep CNN features.

### 2.1. Deep Metric Learning

DML has been a long-standing research hotspot in improving the performance of image retrieval [42,43,44,45,46,52]. There are two different research direction of DML which are clustering-based and pair-based structured losses. We would like to give some detail introduction as follows.

#### 2.1.1. Clustering-Based Structured Loss

The clustering-based structured losses aim to learn a discriminative embedding space by optimizing clustering metric and are applied in abundant fields of computer vision like face recognition [53,54] and fine-grained image retrieval (FGIR) [55,56]. Clustering loss [57] utilizes the structured prediction framework to realize clustering with higher score for ground truth than others. The quality of clustering would be measured by normalized mutual information (NMI) [58]. Center loss [54] suggested to learn a center for each category by compensating for softmax loss and obtain an appreciable performance in face recognition. The triple-center loss (TCL) [59] was proposed to learn a center for each category and separate the cluster centers and their relevant samples from different categories. To enhance the performance of FGIR, centralized ranking loss (CRL) [55] was proposed aiming to optimize centers and enlarge the compactness and separability of intraclass and interclass samples. Later, decorrelated global-aware centralized loss (DGCRL) [56] was proposed to optimize the center space by utilizing Gram-Schmidt independent operation and enhance the clustering result by combining softmax loss. However, all these clustering-based structured losses consume costly in computing and are hard to optimize. Moreover, these losses fail to make full use of the sample relationships which might contain meaningful information for learning a discriminative space.

#### 2.1.2. Pair-Based Structured Loss

As a mass of structured losses [41,42,43,44,45,46,47] have obtained appreciable effectiveness in training networks to learn discriminative embedding features, we would like to make a brief review on the development of pair-based structured loss.

Contrastive loss [41] builds positive and negative sample pairs according to their labels as $\left\{\left(x_{a},\;x_{k}\right),y_{ak}\right\}$ and exploits these constructed pairs to learn a discriminative embedding space by minimizing the distance of positive sample pairs and increasing the distance of negative sample pairs larger than a given threshold m. And the loss function is defined as follows:

(1) $$L_{con}\left(x_{a},x_{k}\right)=\frac{1}{Q}{\sum_{\left(a,k\right)}^{\frac{Q}{2}}\left(1-y_{ak}\right)\left[m-D_{ak}\right]}_{+}^{2}+y_{ak}D_{ak}^{2}$$

where Q is the volume of samples in training set, $y_{ak}=1$ when a sample pair $\left(x_{a},\;x_{k}\right)$ with the same label, and $y_{ak}=0$ when a sample pair $\left(x_{a},\;x_{k}\right)$ with different label. The parameter m is a margin used to limit the distance of negative sample pairs, $D_{ak}$ indicates the Euclidean distance of a sample pair $\left(x_{a},\;x_{k}\right)$ and is defined formularly as $D_{ak}=\|f\left(x_{a}\right)-f{\left(x_{k}\right)\|}_{2}$, and f(·) means the deep feature extracted from the network. ${\left[\cdot \right]}_{+}$ is hinge loss which is to limit the values to be positive.

From Equation (1), we could find that this loss function treats positive and negative pairs equally and fails to take into account the difference between positive and negative sample pairs. As it constructs all samples into pairs locally in training set, it might get fall into local optimum and result in slow convergence.

Triplet loss [42] utilizes abundant triplets to learn a discriminative embedding space to force positive sample pairs closer than negative ones with a given margin m. Each triplet is made up of an anchor sample, a positive sample with the same label to the anchor and a negative sample with different labels to the anchor. To be specific, we denote a triplet as $\left\{\left(x_{a},x_{p},x_{n}\right)\right\}$, $x_{a},x_{p}$ and $x_{n}$ indicate the anchor, positive and negative sample separately. The loss is defined as:

(2) $$L_{trp}\left(x_{a},x_{p},x_{n}\right)=\frac{1}{\left|T\right|}\sum_{\left(x_{a},x_{p},x_{n}\right)\in T}{\left[D_{ap}^{2}-D_{an}^{2}+m\right]}_{+}$$

where T means the collection of triplets, $x_{a},x_{p}$ and $x_{n}$ are the index of anchor, positive and negative samples severally and |T| is the volume of triplets set. $D_{ap}=\|f\left(x_{a}\right)-f{\left(x_{p}\right)\|}_{2}$ and $D_{an}=\|f\left(x_{a}\right)-f{\left(x_{n}\right)\|}_{2}$ denote the Euclidean distance of positive and negative pairs respectively. And f(·) means the deep feature extracted from the network. ${\left[\cdot \right]}_{+}$ is hinge loss which is to limit the values to be positive.

We could learn from Equation (2) that triplet loss does not consider the difference between positive and negative sample pairs which is important for identifying the pairs with more information. Although it takes the relationship between positive and negative pairs into consideration, the rate of convergence is still slow and might struck in local optimal as this loss encode the samples in a training set to triplets set which fails to make full use of sample pairs inside the training set globally.

N-pairs loss [43] takes advantage of the structured information between positive and multiple negative sample pairs in the training mini-batch to learn an effective embedding space. This loss function enhances the triplet loss by training the network with more negative sample pairs and the negative pairs are selected from all negative pairs of other categories. i.e., selecting one sample pair randomly per category. The N-pairs loss is defined as:

(3) $$L_{N-pairs}{\left\{\left(x_{a},x_{p}\right)\right\}}_{a=1}^{Q}=\frac{1}{Q}\sum_{a=1}^{Q}\log\left\{1+\sum_{n\left\{x_{n}|y_{n}\neq y_{a}\right\}}^{Q-1}e^{\left(S_{an}-S_{ap}\right)}\right\}$$

where Q is the number of categories in a training set, and ${\left\{\left(x_{a},x_{p}\right)\right\}}_{a=1}^{N}$ denote N sample pairs which are selected from N different categories, i.e., $x_{a}$ and $x_{p}$ are anchor and its positive sample for a certain category respectively; $\left\{x_{n}|y_{n}\neq y_{a}\right\}$ denotes negative samples for the current anchor; $y_{n}$ and $y_{a}$ denote the labels of $x_{n}$ and $x_{a}$. $S_{ap}=\langle f\left(x_{a}\right),f\left(x_{p}\right)\rangle$ and $S_{an}=\langle f\left(x_{a}\right),f(x_{n})\rangle$ are dot product of positive and negative pairs respectively. The f(·) is the feature representation of an instance.

However, this loss fails to take the difference between negative and positive pairs and neglects some structured information inside the training set. Furthermore, it only selects one positive pair randomly for per class which could lose some significant information during training.

Lifted structured loss [44] was proposed to meet the challenge of local encoding by make full use of information among all the samples in a training batch. It aims to learn an effective embedding space by considering all negative sample pairs of an anchor and encourage the distance of positive pair as small as possible and force the distances of all negative pairs larger than a threshold m. Lifted structured loss is defined as:

(4) $$L_{Lifted}\left(x_{a},x_{p},x_{n}\right)=\frac{1}{2\left|P\right|}{\sum_{\left(x_{a},x_{p}\right)\in P}\left[D_{ap}+\log\left(\sum_{\left(x_{a},x_{n}\right)\in N}e^{m-D_{an}}+\sum_{\left(x_{p},x_{k}\right)\in N}e^{m-D_{pk}}\right)\right]}_{+}$$

where $x_{a}$ and $x_{p}$ are anchor and positive samples respectively and $x_{n}$ and $x_{k}$ are both negative samples, P and N indicate the sets of positive and negative pairs respectively and the |P| is amount of P. $D_{ap}$ is the Euclidean distance of positive pair. $D_{an}$ and $D_{pk}$ are Euclidean distances of negative pairs.

We could learn from Equation (4) that the lifted structured loss makes full use of the relationship between positive and negative sample pairs by constructing the hardest triplet with taking all negative pairs into consideration. However, it fails to keep the structured distribution inside the training set and still fails to realize global optimization as it is a form of hinge loss.

Ranked list loss [46] was proposed to restrict all positive samples into a given hypersphere with diameter as α−m and impel distance of negative sample pairs larger than a fixed threshold α. To be specific, this loss aims at learning a more discriminative embedding space where could separate positive and negative sample set by a margin m and it utilizes a weighting strategy to consider the difference of negative sample pairs:

(5) $$L_{RLL}{\left\{\left(x_{a},x_{p},x_{n}\right)\right\}}_{a=1}^{Q}=\frac{1}{Q}\sum_{a=1}^{Q}\left\{\begin{array}{l} \frac{1}{\left|P_{a}\right|}\sum_{\left(x_{a},x_{p}\right)\in P_{a}}{\left[D_{ap}-\left(\alpha -m\right)\right]}_{+}+\\ \frac{1}{\left|N_{a}\right|}\sum_{\left(x_{a},x_{n}\right)\in N_{a}}\frac{e^{\beta \left(\alpha -D_{an}\right)}}{\sum_{\left(x_{a},x_{n}\right)\in N_{a}}e^{T\left(\alpha -D_{an}\right)}}{\left[\alpha -D_{an}\right]}_{+} \end{array}\right\}$$

where $x_{a},x_{p}$ and $x_{n}$ denote anchor, positive and negative samples respectively and Q is the volume of a training set. $P_{a}$ and $N_{a}$ are the sets of positive and negative pairs for an anchor $x_{a}$. $D_{ap}$ and $D_{an}$ are Euclidean distances of positive and negative pairs respectively which have been described above. β is a parameter which is used to reflect the degree of negative samples during weighting.

We could know that the ranked list loss has obtained an appreciable performance in multiple image retrieval tasks. However, it does not take the relationship between positive and negative sample pairs which is important to enhance the robustness and distinctiveness of network. Moreover, as it utilizes hinge function to optimize this loss which might be easy to lead to local optimum, the performance still couldn’t meet our demands in RSIR.

To solve the limitations of existing DML methods, we propose to exploit the softmax function instead of the commonly used hinge function in our loss function to realize global optimization. Furthermore, we make full use of the structured information and maintain the inner similarities structure by setting positive and negative boundary for sample pairs during training stage.

#### 2.1.3. Informative Pairs Mining

During the training stage, there are vast numbers of less informative sample pairs which might slow down convergence and result in a local optimum. It is significant to design a superior pairs mining scheme for training efficiency. There are many excellent studies on informative pairs mining scheme design [43,44,45,46,53,60]. A semi-hard mining strategy was proposed to sample a handful of triplets which contain a negative pair farther than positive one in FaceNet [53]. A more effective pairs mining framework was proposed to select hard samples from the database for training [60]. Sohn et al. proposed hard negative categories mining to collect more informative samples for training the network globally [43]. Song et al. proposed to select harder negative samples to optimize lifted structured loss [44]. Wang.et al. provided a simple pairs mining strategy which select the sample pairs in violation of distance restriction [46]. Wang. et al. designed a more effective pairs mining scheme to obtain more excellent performance which take the relationship between positive and negative sample pairs into consideration [45]. In this paper, we propose to utilize the pairs mining scheme proposed in [45] to realize more informative sample pairs mining and improve the performance of RSIR.

### 2.2. The Development of RSIR Task

In the last few decades, the task of RSIR has been received extensive attention from researchers and the wide studies have spawned a whole bunch of elegant methods. We would like to give some introduction on the methods for RSIR in terms of traditional handcrafted representation and deep representation methods. Moreover, we introduce some works related to the RSIR under DML.

In the initial time, researchers tended to extract textural features for remote sensing image classification [11,61]. Datcu et al. presented a special pipeline for the task of RSIR and proposed to utilize the model of Bayesian inference to capture spatial information for features extraction [62]. And at the same time, Schroder et al. proposed to exploit Gibbs-Markov random fields (GMRF) which could be used to capture spatial information to extract features [63]. Daschiel et al. suggested to utilize hierarchical Bayesian model to extract feature descriptors and these features are clustered by the dyadic k-means methods [64]. With the development of general image retrieval, Shyu et al. proposed a comprehensive framework defined as geospatial information retrieval and indexing system (GeoIRIS) for RSIR based on CBIR [65]. This system could be used to automatically extract features, mine visual content for remote sensing images and realize fast retrieval by indexing from database. The features are mainly based on patch which could be helpful to maintain some local information. And to enhance the retrieval precision, they extract various visual features including general features like spectral and texture features and anthropogenic features like linear and object features. However, these methods based on global visual features mentioned above are hard to maintain invariance to translation, occlusion and translation. With the introduction of SIFT descriptors [15], Yang et al. proposed to utilize BoW to encode SIFT features extracted from remote sensing images and the experiments have demonstrated that the method based on local features could be superior than global visual features [66]. Later, more works tend to use local features to realize efficient retrieval [16,67]. More recently, there are some studies that tend to utilize features extracted from remote sensing images to retrieve local climate zones [68,69]. However, these handcrafted features fail to extract richer information from remote sensing images as their limited descriptive ability.

With the successful application of deep learning in general image retrieval task, deep features extracted from CNN are gradually exploited to achieve more appreciable performance in RSIR [10,70,71]. Bai et al. proposed to map deep features into a BoW space [70]. Li et al. proposed to combine handcrafted features with deep features to produce more effective features for RSIR [71]. Ge et al. tended to combine and compress deep features extracted from pre-trained CNNs to enhance the descriptive power of features [10]. All these methods mentioned above have made great contributions on improving the performance of RSIR. However, these methods are mainly based on pre-trained networkd which might not be suitable for the task of RSIR. To further improve the performance, recent works tend to concentrate on fine-tuning the pre-trained network for RSIR [32,49,50,72,73]. Li et al. proposed to fine-tune a pre-trained CNN to learn more effective feature descriptors and the network is trained on remote sensing datasets [73]. Li et al. made a try on combining deep features learning network and deep hashing network together to develop a novel deep hash neural network which is trained in an end-to-end manner for RSIR [72]. Tang et al. proposed to utilize deep BOW (DBOW) to learn deep features based on multiple patches in an unsupervised way [50]. Wei et al. presented a multi-task learning network which is connected with a novel attention model and proposed to utilize center loss for network training [32]. Raffaele et al. proposed to conduct the aggregation operation of VLAD on the local deep features extracted from fine-tuned CNNs with two different attention mechanisms to eliminate the influence of irrelated background [49].

More and more elegant works prefer to apply DML in the field of remote sensing images to enhance the effectiveness of RSIR [30,33,34,35,36,37]. Roy et al. proposed a metric and hash-code learning network (MHCLN) which could be used to learn semantic embedding space and produce hash codes at the same time [33]. It aims to realize accurate and fast retrieval in the task of RSIR. Cao et al. presented a novel triplet deep metric learning network for RSIR, the remote sensing images are embedded into the learned embedding space where the positive sample pairs closer and negative ones far away from each other [34]. Subhanker et al. presented a novel hashing framework which is based on metric learning [35]. Most existing DML methods for RSIR are mainly based on triplet loss which is limited with the local optimization and inadequate use of sample pairs. In this paper, we investigate the effectiveness of RSIR when applying more superior DML methods. Furthermore, we propose a more efficient loss function to learn a discriminative embedding space for remote sensing images to achieve elegant performance for the task of RSIR.

## 3. The Proposed Approach

In this section, we give some detailed descriptions about our proposed method which includes five parts. Firstly, we give the problem definition on the task of RSIR. In Section 3.2, Section 3.3 and Section 3.4, we describe our proposed loss function and the optimization process in detail.

### 3.1. Problem Definition

We denote the input images as $x=\left\{x_{1},\ldots ,x_{a},\ldots ,x_{Q}\right\}$ for a training set. There are C classes in a training set and we denote the labels for n input images as $y=\left\{y_{1},\ldots ,y_{a},\ldots ,y_{n}\right\}$ where $y_{a}\in \left\{1,\ldots ,c,\ldots ,C\right\}$, particularly. There is only one label $y_{a}$ for an input image $x_{a}$. The input images x are projected onto a d-dimension embedding space by utilizing a deep neural network with batch normalization which could be indicated as f(x,θ). To be specific, f is the deep mapping function of the network and θ is a set of parameters need to be optimized of the mapping function f. In this paper, we use inner product $S_{ak}$ to measure the similarity of any two images $\left(x_{a},x_{k}\right)$ during the training and testing phases and we denote the similarity metric as $S_{ak}=\langle f\left(x_{a};\theta \right),f(x_{k};\theta )\rangle$. As we exploit all samples in a training batch as anchor and compute the similarity of all samples with an anchor, we could denote the similarities of a training batch as an n×n matrix S and use $S_{ak}$ to represent the element at (a,k).

### 3.2. Global Lifted Structured Loss

As described in Section 2.1.2, the lifted structured loss utilizes a set of triplets for training, which is dynamically constructed by considering all sample pairs except the positive pair as negatives. It takes all negative pairs but only one positive pair into consideration for each triplet. To meet this limitation, a more generative loss function is proposed to learn a more discriminative embedding space by considering all positive pairs in a training batch in person re-ID [74]. The loss is defined as:

(6) $$L_{GeL}\left(x\right)=\frac{1}{Q}{\sum_{a=1}^{Q}\left[\log\sum_{y_{k}=y_{a}}e^{D_{ak}}+\log\sum_{y_{k}\neq y_{a}}e^{m-D_{ak}}\right]}_{+}$$

There are two parts in this loss function. The distance between positive and negative pairs is denoted as $D_{ak}=\|f\left(x_{a},\theta \right)-f{\left(x_{k},\theta \right)\|}_{2}$ and m is a margin. In our paper, we utilize inner product to measure similarity. It’s noted that the Euclidean distance could be converted to inner product as follows:

(7) $$\begin{array}{l} {\left\|f\left(x_{a},\theta \right)-f\left(x_{k},\theta \right)\right\|}_{2}\\ ={\left\|f\left(x_{a},\theta \right)\right\|}_{2}+{\left\|f\left(x_{k},\theta \right)\right\|}_{2}-2f{\left(x_{a},\theta \right)}^{T}f\left(x_{k},\theta \right)\\ =A-2f{\left(x_{a},\theta \right)}^{T}f\left(x_{k},\theta \right) \end{array}$$

where A is a constant. We could learn from Equation (7) that the Euclidean distance and inner product is inversely proportional to each other. In our paper, we exploit inner product to measure similarities. We recompute the generative lifted structured loss to inner product and we denote the formula as:

(8) $$L_{GeLS}\left(x\right)=\frac{1}{Q}{\sum_{a=1}^{Q}\left[\log\sum_{y_{k}=y_{a}}e^{-S_{ak}}+\log\sum_{y_{k}\neq y_{a}}e^{\mu +S_{ak}}\right]}_{+}$$

where μ is a given margin. However, the generative lifted structured loss still fails to solve the limitation of encoding pairs locally which might result in local optimum. To breakthrough this limitation, we use the softmax loss to realize globally optimizing. As the softmax loss is used to deal with the task of classification, we here take our task as a classification of positive and negative similarity. The formula is defined as:

(9) $$L_{soft}\left(x_{a},x_{k}\right)=-\log\frac{e^{S_{ak}}}{\sum_{\left(x_{a},x_{k}\right)}e^{S_{ak}}}$$

As our target is to increase the similarities of positive pairs (i.e., draw the distance close for positive pairs) and reduce the similarities of negative pairs (i.e., make the distance further for negative pairs), we could take the limit for the similarities for positive and negative pairs. Specifically, we assume the positive and negative similarities (measured by inner product) are infinitely close to +1 and −1 respectively (i.e., positive and negative distances (measured by Euclidean distance) are 0 and +∞ respectively) which means that the numerator in Equation (9) is a constant. And we give definition of the probabilities for positive and negative similarities to an anchor as $R_{y_{k}=y_{a}}=A_{1}/\sum_{y_{k}=y_{a}}e^{-S_{ak}}$ and $R_{y_{k}\neq y_{a}}=A_{2}/\sum_{y_{k}\neq y_{a}}e^{\mu +S_{ak}}$. $A_{1}$ and $A_{2}$ are both constant. We combine the softmax loss with the generative lifted structured loss as:

(10) $$L_{GLS}\left(x\right)=\frac{1}{Q}\sum_{a=1}^{Q}\left\{\log\sum_{y_{k}=y_{a}}e^{-S_{ak}}+\log\sum_{y_{k}\neq y_{a}}e^{\left(\mu +S_{ak}\right)}\right\}$$

This global lifted structured loss could be likely to learn a discriminative embedding space globally. However, it still fails to eliminate the impact of less informative sample pairs and keep the sample pairs distribution inside the training batch. To achieve better performance in RSIR, we propose to use an efficient pairs mining strategy to select sample pairs with richer information and propose a global optimal structured loss which could increase the intraclass compactness and maintain the distribution of the selected sample pairs at the same time for network model training. We would like to give the detailed description about our mining scheme and global optimal structured loss.

### 3.3. Global Optimal Structured Loss

For the task of RSIR, our target is to increase intraclass compactness and interclass sparsity. However, the proposed global lifted structured loss described in Section 3.2 fails to keep the distribution of sample pairs inside the selected sample pairs set. In our paper, we propose a novel global optimized structured loss which is used to learn an efficient and discriminative embedding space. It aims to limit sample pairs with the same class label (positive sample pairs) within a hypersphere with diameter of (α−m). The fixed boundary could be important to maintain similarity distribution of the selected positive pairs for each category. And simultaneously all negative sample pairs could be pushed away from a fixed boundary α, the positive and negative sample pairs could be separated by a margin m.

We intend to use the pairs mining strategy described in [45], which exploits the hardest negative pair (with the largest similarity among all negative pairs) to mine informative positive pairs and similarly sample negative pairs with richer information by considering the hardest positive pair (with the smallest similarity among all positive pairs). In other word, for an anchor $x_{a}$, we sample the informative positive and negative pairs according to the following two formulas. The informative positive and negative pairs sets are denoted as $P_{a}$ and $N_{a}$ respectively. The formulas are defined as:

(11) Sap+<Sakyk≠yamax+ϵ

(12) San−>Sakyk=yamin−ϵ

where ϵ=0.1. From Equation (11), we could know that we select the positive pair $\left(x_{a},x_{p}\right)$ as an element of $P_{a}$ by comparing its similarity with the hardest positive similarity. And we could learn from Equation (12) that the negative pair $\left(x_{a},x_{n}\right)$ is selected as an element of $N_{a}$ by comparing its similarity with the hardest positive similarity. And ϵ is a hyper-parameter used to control the scope of informative sample pairs.

To realize the target of pulling the mined positive pairs as close as possible and keeping the similarity distribution of each class sample pairs (positive pairs) simultaneously, we increase their similarities and force them to be larger than the positive boundary (α−m) by minimizing the positive part of our proposed loss function. It is defined as:

(13) $$L_{p}\left(x_{a}\right)=\log\sum_{\left(x_{a},x_{p}\right)\in P_{a}}e^{-\left(S_{ap}^{+}+\left(\alpha -m\right)\right)}$$

Similarly, to achieve the goal of pushing the mined negative sample pairs far away from positive ones and realize the separation of positive and negative sample pairs, we propose to decrease the negative similarities and impel them to be smaller than the negative boundary α by minimizing the negative part of our proposed loss function. We define this as:

(14) $$L_{N}\left(x_{a}\right)=\log\sum_{\left(x_{a},x_{n}\right)\in N_{a}}e^{\left(S_{an}^{-}+\alpha \right)}$$

For our proposed global optimal structured loss, we integrate the two part of minimization objectives and optimize them jointly. And as there is difference between positive and negative sample pairs, we utilize two different hyper-parameters $\beta_{1}$ and $\beta_{2}$. Our proposed loss is represented as:

(15) $$L_{GOS}\left(x_{a}\right)=\frac{1}{\beta_{1}}\log\sum_{\left(x_{a},x_{p}\right)\in P_{a}}e^{-\beta_{1}\left(S_{ap}^{+}+\left(\alpha -m\right)\right)}+\frac{1}{\beta_{2}}\log\sum_{\left(x_{a},x_{n}\right)\in N_{a}}e^{\beta_{2}\left(S_{an}^{-}+\alpha \right)}$$

where $\beta_{1}=2$, $\beta_{2}=50$. This global optimal lifted structured loss could be likely to pay more attention on the positive and negative pairs with more information, which would be helpful to further improve the performance and effectiveness of RSIR task.

To make full use of sample pairs among the mini-batch, we treat all images in a mini-batch as an anchor and the rest of images except the current anchor as gallery iteratively. And we would like to define the loss function for a mini-batch as follows:

(16) $$L_{GOS}\left(x\right)=\frac{1}{Q}\sum_{a=1}^{Q}\left\{\frac{1}{\beta_{1}}\log\sum_{\left(x_{a},x_{p}\right)\in P_{a}}e^{-\beta_{1}\left(S_{ap}^{+}+\left(\alpha -m\right)\right)}+\frac{1}{\beta_{2}}\log\sum_{\left(x_{a},x_{n}\right)\in N_{a}}e^{\beta_{2}\left(S_{an}^{-}+\alpha \right)}\right\}$$

After the loss function has been defined, the network parameters could be learned by Back-Propagation. We minimize the $L_{GOS}$ with gradient descent optimization by conducting online iterative pairs mining and loss calculation in the form of matrix. We could compute the loss of deep features in training set f(x,θ) by utilizing Equation (16). And its gradient of with respect to f(x,θ) could be denoted as:

(17) $$\begin{array}{c} \frac{\partial L_{GOS}\left(x\right)}{\partial f\left(x,\theta \right)}=\frac{1}{Q}\sum_{a=1}^{Q}\frac{\partial L_{GOS}\left(x_{a}\right)}{\partial f\left(x,\theta \right)}\\ =\frac{1}{Q}\sum_{a=1}^{Q}\left(-\sum_{\left(x_{a},x_{j}\right)\in P_{a}}\frac{1}{\sum_{\left(x_{a},x_{p}\right)\in P_{a}}e^{-\beta_{1}\left(S_{ap}^{+}-S_{aj}^{+}\right)}}\frac{\partial S_{aj}^{+}}{\partial f\left(x,\theta \right)}+\sum_{\left(x_{a},x_{j}\right)\in N_{a}}\frac{1}{\sum_{\left(x_{a},x_{n}\right)\in N_{a}}e^{\beta_{2}\left(S_{an}^{-}-S_{aj}^{-}\right)}}\frac{\partial S_{aj}^{-}}{\partial f\left(x,\theta \right)}\right)\\ =\frac{1}{Q}\sum_{a=1}^{Q}\left(-\sum_{\left(x_{a},x_{j}\right)\in P_{a}}w_{aj}^{+}\frac{\partial S_{aj}^{+}}{\partial f\left(x,\theta \right)}+\sum_{\left(x_{a},x_{j}\right)\in N_{a}}w_{aj}^{-}\frac{\partial S_{aj}^{-}}{\partial f\left(x,\theta \right)}\right) \end{array}$$

(18) $$w_{aj}^{+}=\frac{1}{\sum_{\left(x_{a},x_{p}\right)\in P_{a}}e^{-\beta_{1}\left(S_{ap}^{+}-S_{aj}^{+}\right)}}$$

(19) $$w_{aj}^{-}=\frac{1}{\sum_{\left(x_{a},x_{n}\right)\in N_{a}}e^{\beta_{2}\left(S_{an}^{-}-S_{aj}^{-}\right)}}$$

In Equation (17), we could regard $w_{aj}^{+}$ and $w_{aj}^{-}$ as the weight for positive and negative similarity respectively. The network parameter update is determined by both positive and negative similarity, and the loss of positive (negative) similarity is used reflect intraclass compactness (interclass sparsity). We give the optimization process in Algorithm 1.
**Algorithm 1:** Global Optimal Structured Loss on a mini-batch.1: mini-batch default: The size of every mini-batch is B, the number of categories is E, and there are M instances in every category. 2: hyper-parameters default: The scope constraint for pairs mining is ϵ, the negative boundary is α, the margin between positive and negative boundary is m, the positive and negative temperature $\beta_{1}$ and $\beta_{2}$. 3: Input: $=\;\left\{x_{1},\ldots ,x_{a},\ldots ,x_{Q}\right\},$
$y=\left\{y_{1},\ldots ,y_{a},\ldots ,y_{Q}\right\}$, the features are extracted by f(x,θ). 4: Output: Updated network parameters f(x,θ). 5: The forward propagation: for a=1,…,n do feed forward $x_{a}$ into network and output the deep feature $f\left(x_{a},\theta \right)$. 6: Similarity matrix calculation: calculating all similarities in the mini-batch according to the formula $S_{ak}=\langle f\left(x_{a};\theta \right),f(x_{k};\theta )\rangle$ to obtain an n×n matrix S. 7: Global Optimal Structured Loss computation: 8:   For a=1,…,Q do 9:      Construct informative positive pairs set $P_{a}$ for anchor $x_{a}$ as Equation (11) 10:      Construct informative negative pairs set $N_{a}$ for anchor $x_{a}$ as Equation (12) 11:      Calculate $L_{P}$ as Equation (13) for the sampled positive pairs 12:      Calculate $L_{N}$ as Equation (14) for the sampled negative pairs 13:      Calculate $L_{GOS}\left(x_{a}\right)$ as Equation (15) for an anchor $x_{a}$ 14:   end for 15:   calculate $L_{GOS}\left(x\right)$ as Equation (16) for a mini-batch. 16: Backpropagation gradient and network parameters f(x,θ) update: 17:   $f\left(x,\theta \right)=f\left(x,\theta \right)-w\frac{\partial L_{GOS}\left(x\right)}{\partial f\left(x,\theta \right)}$.

### 3.4. RSIR Framework Based on Global Optimal Structured Loss

**Figure 2:**
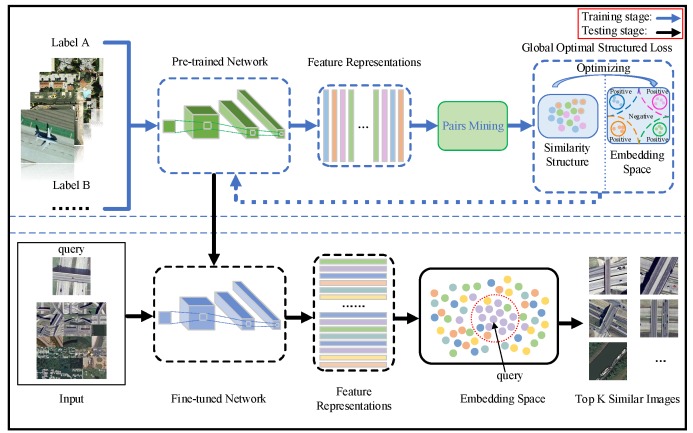
The RSIR framework based on the global optimal structured loss. The upper part denotes training stage and we fine-tune the pre-trained network with our global optimal structured loss. We utilize the fine-tuned network for more discriminative feature representations extraction. The bottom part is testing stage. The query image and the testing set would be input in the fine-tuned network, and the top K similar images would be returned.

In this section, we illustrate the RSIR framework based on our proposed global optimal structured loss which contains the stages of training and testing. We present this framework in Figure 2. 

During the training stage, we utilize our proposed method to fine-tune the pre-trained network and we have illustrated the optimization process in detail in Section 3.4. We exploit the pre-trained network to extract deep features and generate a feature matrix for a training mini-batch. We perform similarity calculation on feature matrix by inner product operation to obtain a similarity matrix with size Q×Q. And then we utilize our proposed global optimal structured loss to optimize the embedding space by increasing the similarity of positive sample pairs and reducing the similarity of negative ones which are selected by using a superior pairs mining scheme. The optimal embedding space could be efficient to force positive pairs more compact within a fixed hypersphere and impel different class pairs apart away from each other with a given margin. At the stage of testing, we utilize the fine-tuned network to extract deep features which could be more discriminative. We conduct the similarity computing operation (inner product) on the feature matrix to return a similarity matrix for a test set. Lastly, the top K similar remote sensing images would be returned according the values of similarities for each query.

## 4. Experiments and Discussion

In this section, we represent some details about the implementation of our experiments and verify the effectiveness of our proposed method by conducting experiments on different remote sensing datasets.

### 4.1. Experimental Setup

#### 4.1.1. Experimental Implementation

We perform the experiments on Ubuntu 16.04 with a single RTX 1080 Ti GPU and 64 GB RAM. We implement our method by using Pytorch. The Inception network with batch normalization [75] which is pre-trained on ILSVRC 2012-CLS [76] would serve as our initial network. Moreover, during training, a FC layer is added on the top of our initial network and it is behind the global pooling layer. We utilize Adam as optimizer to implement our experiments. The learning rate is set to $1e^{-5}$ during training for our all experiments; the training process would be converged at 600 epochs. We use retrieval precision [50] to report the experimental results. The retrieval precision could be defined as TP/R, where TP is the number of images belong to the same category and R is the amount of returned images (candidates) for a query q. We select all images in the test set as query images and the final results which would be denoted as AveP:

(20) $$AveP=\frac{1}{\left|Q\right|}\sum_{q=1}^{\left|Q\right|}\frac{TP}{R}$$

where |Q| means the volume of query images in the test set, R denotes the returned images for a query q, TP is the number of true positive images for a query q. And in our paper, we only return the top 20 retrieval images (candidates) by following the setting in DBOW [50].

#### 4.1.2. Datasets and Training

Datasets. We perform our experiments on four kinds of different remote sensing databases: UCMerced Land Use [16,66], Satellite Remote Sensing Image Database [77], Google Image Dataset of SIRI-WHU [17,19,78] and NWPU-RESISSC45 [1]. We would like to give an introduction to these benchmark databases as follows:

UCMerced Land Use [16,66] is collected from large amount of images download from the United States Geological Survey (USGS) by the team at the University of California Merced. This dataset is commonly used in tasks of retrieval and classification in the field of RSIA. UCMerced Land Use includes 21 geographic categories and there are 100 remote sensing images per category, the size of an image is 256×256 pixel with 0.3 m spatial resolution. We denote this dataset as UCMD in the remaining parts of this section.

Satellite Remote Sensing Image Database [77] contains 3000 remote sensing images of 256×256 pixel and the spatial resolution of each pixel is 0.5 m. There are 20 geographic categories labeled manually and each category includes 150 images. We denote this dataset as SATREM for convenience in the remainder of this section.

Google Image Dataset of SIRI-WHU [17,19,78] contains 2400 remote sensing images with size of 200×200 pixel and the spatial resolution of each pixel is 2 m. This dataset contains 12 geographic categories and there are 200 images in a certain category. As a matter of convenience, we denote this dataset as SIRI in experiments and discussion.

NWPU-RESISSC45 [1] is collected from Google Earth and is a large-scale remote sensing dataset. There are 31,500 remote sensing images totally and the size of image is 256×256 pixel. The spatial resolution of them varies from 30 to 0.2 m. This dataset contains 45 geographic categories and each category owns 700 remote sensing images. In order to facilitate the discussion in the remaining parts of this section, we indicate this dataset as NWPU.

Training setting. By following the data split protocol used in DBOW [50], we divide the training and testing set on a scale of 4:1 for each dataset. We crop the size of all input images to 224×224. In order to avoid overfitting during training, the data augmentation operation of random crop with random horizontal mirroring is applied in our experiments. As for testing stage, we utilize single center crop to realize data augmentation. During training, we set the size of every mini-batch as B. A mini-batch consists of a certain amount of random geographic categories, and we sample M random images from each geographic category for training. We set M=5 in all experiments by following the work of Wang et al. [45]. According to the analysis described in the section of ablation study, we set the hyper-parameters mentioned in Section 3 as $\beta_{1}=2,\;\beta_{2}=50,\epsilon =0.1,\alpha =0.8,m=0.5$ in following experiments.

### 4.2. Comparision with the Baselines

Baselines. Tang and Raffaele successively performed comprehension comparisons on multiple systems [49,50]. We record the method proposed by Tang et al. as DBOW [50] and the method proposed by Raffaele et al. as ADLF [49] for convenience. Besides the DBOW and ADLF, we also select other three excellent works provided in DBOW and ADLF as baselines for comparison. The baselines could be introduced in detail in Table 1. For DN7 [28] and DN8 [28], the results are obtained by using the DN features extracted from the 7th and 8th fully connected layers in DBOW. For ResNet50, the result is obtained by using the VLAD encodings following ResNet 50 [51]. We would directly utilize the obtained results in their works as reference for comparisons. To verify the superiority of our proposed global optimal structured loss, we conduct a set of experiments on four different remote sensing datasets. We compare our proposed method with the baselines in the task of RSIR.

As mentioned in Section 3, we fine-tune the network with our proposed global optimal structured loss. We utilize the features extracted from the fine-tuned network for four different remote sensing datasets to realize the task of RSIR and perform a comparison with the baselines mentioned above. We set the embedding size to 512 and batch size to 40 in our experiments. Herein, we denote our proposed global optimal structured loss with pairs mining strategy as GOSLm. We present the results in Table 2.

We could conclude from Table 2 that our global optimal structured loss with pairs mining strategy obtains the state-of-the-art results on the datasets of SIRI and NMPU. The *AveP* (%) outperforms the DBOW by 4% (from 92.6% to 96.6%) on SIRI and obtains the improvement of 4.6% (from 85.7% to 90.3%) on NMPU over ADLF. As for the datasets of UCMD and SATREM, we achieved the second-best performance with the *AveP* (%) is 85.8% and 91.1% respectively. While the best results on UCMD is obtained by ADLF which is with the post-processing of query expansion (QE), but on the remaining three datasets, our method would achieve stronger performance than ADLF. DBOW obtains the best performance on SATREM. However, our proposed method would outperform the DBOW on the remaining three datasets. Furthermore, it’s worth noting that we conduct our experiments with raw feature representations without any post-processing operations like whitening, re-ranking and QE. We could learn that our proposed method shows great effectiveness in the field of RSIR and could obtain the state-of-the-art results on commonly used remote sensing datasets. To further investigate the effectiveness of our proposed method, we would like to show the precisions of the different geographic categories in the four remote sensing datasets in Table 3, Table 4, Table 5 and Table 6 and the best results would be highlighted in bold. We utilize the top 20 retrieval images to compute the precision results for per geographic category.

We could learn from Table 3 that our method achieves a marked improvement in nearly half of categories. Specifically, our proposed method makes the most prominent promotion on “Golf” and “Sparse” with the increase of 7% (from 85% to 92%) and 12% (from 79% to 91%). Moreover, we also make some small promotion on some categories. Specifically, the proposed method increases the precision by 1% (from 94% to 95%) over DN7 on “Agriculture”, 3% (from 87% to 90%) over DBOW on “Baseball”, 2% (from 93% to 95%) over DBOW on “Storage” and 1% (from 94% to 95%) over DBOW and ADLF on “Tennis”. However, the weaker performance is obtained on other categories and we would like to report the results as follows. The precisions are 82%, 92%, 78%, 95%, 95%, 83%, 95%, 80%, 78% and 91% on the categories of “Airplane”, “Beach”, “Buildings”, “Chaparral”, “Forest”, “Freeway”, “Harbor”, “Intersection”, “Overpass”, “Runway” respectively which are about on average level. We also come in second place on “Mobile”, “Parking” and “River” with the precisions are 80%, 95% and 86% respectively. And our proposed method obtains the worst results on “Dense” and “Medium-density” with the precision of 55% and 59% respectively. We make a further research on the retrieval results and it turns out that our method is confused by the images belong to “Dense” with “Medium-density”, “Mobile” and “Buildings”. The averages of all precisions on UCMD with our proposed method comes in the second place and the result is 85.8%.

From Table 4, we could know that our method outperforms the state-of-the-art methods on half of the categories in SATREM. Especially, our proposed method could make a great enhancement on the categories of “Airplane”, “Beach”, “Chaparral” and “Ocean”. The precisions on these categories are 100%, 98%, 100% and 100% respectively, which are increased nearly by 4% comparied with the existing best results. We also obtain fine improvements on some categories. Specifically, the precisions are increased by 1% (from 97% to 98%) on “Artificial” and 2% (from 96% to 98%) on “Forest”. Moreover, we obtain the same best results compared with the existing best methods on the categories of “Cloud”, “Harbor” and “Runway” with the precisions of 100%, 98% and 97% respectively. However, our method obtains weaker results on some other categories. We achieve the second-best results on “Agriculture”, “Buildings”, “Road” and “Storage”, the precisions on these categories are reported as 92%, 94%, 90% and 99% respectively. And the results on the categories of “Container”, “Dense”, “Factory”, “Parking” and “Sparse” are mundane and they are mainly on the average level, the precisions on these categories are reported as 92%, 92%, 72%, 88% and 78%. The worst result is obtained on the category of “Medium-density” with the precision of 53%. The further analysis of retrieval results has shown that abundant incorrect images belong to “Building”, “Dense Residential” and “Factory” retrieved for “Medium-density” images. For the average of the precision of all categories in SATREM, we could achieve a competitive result compared with the state-of-the-art results. Our proposed method obtains the second-best result with 91.1%.

The results in Table 5 show that our proposed method achieves the state-of-the-art performance in almost all categories. To be specific, we achieve significant improvements compared with the existing best results on the categories of “Harbor”, “Overpass” and “Park” with the improvement of 9% (from 89% to 98%), 6% (from 94% to 100%) and 10% (from 90% to 100%) respectively. We increase the precision slimly by 1% (from 99% to 100%) over DBOW on “Commercial”, 2% (from 97% to 99%) over DBOW on “Idle”, 2% (from 96% to 98%) over ADLF on “Industrial”, 2% (from 93% to 95%) over DBOW on “Meadow”, 1% (from 97% to 98%) over DBOW on “Residential” and 1% (from 99% to 100%) over ADLF on “Residential”. However, we obtain weaker results on the categories of “Pond” and “River” and the precisions are reported as 96% and 77% which are on the average level. The final *AveP* of all images in SIRI is increased by approximately 4% (from 92.6% to 96.6%). The improvement achieved on dataset of SIRI demonstrates that our method could be more effective and superior than the state-of-the-art methods in processing the task of RSIR.

We could learn from Table 6 that our proposed method promotes the retrieval performance for most of categories in NWPU. Especially, we make significant improvements on many categories. Our method increases the retrieval precision drastically by 11% (from 85% to 96%) over DBOW on “Beach”, 16% (from 80% to 96%) over DBOW on “Ground Track Field”, 17% (from 80% to 97%) over DBOW on “Intersection”, 14% (from 76% to 90%) over DBOW on “River”, 26% (from 69% to 95%) over ADLF on “Ship”, 15% (from 80% to 95%) over ResNet50 on “Tennis Court” and 11% (from 78% to 89%) over ADLF on “Thermal Power Station”. We also achieve ordinary improvements of 5% to 10% on the categories of “Baseball Diamond”, “Basketball Court”, “Overpass”, “Roundabout”, “Sparse Residential”, “Stadium” and “Wetland” and the obtained best precisions on these categories are reported as 93%, 90%, 95%, 95%, 93%, 92% and 85%, respectively.

Moreover, the proposed method makes fine promotions which are less than 5% on the categories of “Freeway”, “Harbor”, “Industrial Area”, “Lake”, “Parking Lot”, “Runway”, “Snowberg” and “Storage Tank” and their precisions are 88%, 99%, 90%, 89%, 98%, 90%, 99% and 98% respectively. On the categories of “Cloud”, “Meadow” and “Sea Ice”, the proposed method obtains the same best results compared with the existing best methods with the retrieval precisions are 98%, 93% and 99%, respectively.

However, we achieve weaker performance on some categories. We achieve the second-best performance on the categories of “Airplane”, “Airport”, “Bridge”, “Chaparral”, “Church”, “Circular Farmland”, “Dense Residential”, “Golf Course”, “Island”, “Medium Residential”, “Mobile Home Park”, “Railway Station”, “Rectangular Farmland” and “Terrace”, the retrieval precisions on these categories are reported as 96%, 90%, 93%, 98%, 64%, 97%, 92%, 96%, 93%, 82%, 94%, 81%, 86% and 89% respectively. The performance on the categories of “Commercial Area”, “Desert”, “Forest”, “Mountain”, “Palace” and “Railway” is on the average level and the retrieval precisions are reported as 78%, 90%, 94%, 86%, 51% and 77% respectively. As for the average precision of all categories, the result is increased from 85.7% to 90.3% with nearly 4.5% enhancement. The results demonstrate the effectiveness and superiority of our proposed method.

### 4.3. Comparison with Multiple DML Methods in the Field of RSIR

As described in Section 2.1.2, there are many proposed elegant DML methods and these methods have achieved appreciable performance in the tasks of general and fine-grained image retrieval. To verify the generalization ability of DML in the task of RSIR, we perform a set of experiments on four datasets with common DML methods of N-pairs loss [43], global lifted structured loss [74], our proposed global optimal structured loss and the latter two methods with pairs mining scheme. For convenience, we denote the global lifted structured loss, N-pairs loss and our global optimal structured loss as GLSL, N-pairs and GOSL respectively. Moreover, we use the subscript m to indicate whether employing our mining scheme. For all these DML methods, we set the embedding size to 512 and batch size at B=40 in our experiments unless otherwise stated. For GLSL, we follow the experimental implementation and training set of our proposed global optimal structured loss with pairs mining scheme and the hyper parameter is set as μ=0.5. And the GLSL_m_ would follow the same setting of GLSL and the hyper parameter of mining scheme is set as ϵ=0.1. As for N-pairs, we follow the experimental implementation and training set of our proposed global optimal structured loss with pairs mining scheme but the batch size and the number of images sampled from each category would be set as B=20 and M=2. We would like to represent the results of *AveP* (%) in Table 7.

We could learn from Table 7 that the task of RSIR could achieve appreciable performance on the public remote sensing datasets with common DML methods. Firstly, we analyze the performance of the methods on UCMD dataset as follows. Our GOSL_m_ achieved the best performance with AveP=85.5% and it outperforms GOSL, GLSL_m_, GLSL and N-pairs by 0.7%, 1.5%, 3.2% and 3.6% respectively. Moreover, we could conclude that the GLSL and our GOSL with pairs mining scheme could increase the *AveP* by 0.7% and 1.7% respectively over the counterparts without pairs mining scheme. Secondly, we make a conclusion on the SATREM dataset according to the results reported in Table 7 as follows. We achieve the best performance (*AveP =* 91.1%) with our GOSL_m_ and it outperforms GLSL_m_ and N-pairs with 3.9% and 5.8% respectively. We could also learn that with pairs mining scheme, the performance of GLSL and GOSL would be promoted by a wide margin. To be specific, GOSL_m_ improves the *AveP* from 86.8% to 91.1% over GOSL and GLSL_m_ improve the *AveP* from 85.1% to 87.2% over GLSL. Thirdly, we analyze the results on SIRI with different DML methods. With the pairs mining scheme, our GOSL_m_ could obtain the best performance with *AveP* =96.6% and outperforms the GOSL with 1.3%. The pairs mining scheme also improves the performance of GLSL from 94.9% to 95.2%. Moreover, the *AveP* of our GOSL_m_ is better than GLSL_m_ and N-pairs. In the end, we analyze the results on NWPU according to the results in Table 7. We achieve the best performance with our proposed GOSL_m_ which is higher than GLSL_m_ and N-pairs by 1.7% and 6.0% respectively. Furthermore, the GLSL_m_ increases the *AveP* by 3.1% over GLSL and the proposed GOSL_m_ increases the *AveP* by 4.5% over GOSL. In brief, our proposed global optimal structured loss with pairs mining scheme could achieve the best performance on the four popular remote sensing datasets. The proposed novel loss is more effective than the common DML methods and the pairs mining scheme could be helpful to further boost the performance of DML methods.

To further study the efficiency of our proposed method, we propose to utilize Recall@K [44] (K = 1, 2, 4, 8, 16, 32) to evaluate the performance of RSIR with these common DML methods and our proposed method. Recall@K is a common metric used in retrieval task which is the average recall scores over all query images in a test set. We perform the experiments on the four remote sensing datasets with the same settings as the first part of this section. The results would be reported in Table 8, Table 9, Table 10 and Table 11.

From Table 8, we could learn that we achieve the best performance with our proposed GOSL_m_ at the metric of Recall@K (K = 1, 2, 4, 8, 16, 32) and the results are reported as Recall@1 = 98.5%, Recalll@2 = 98.8%, Recall@4 = 99.0%, Recall@8 = 99.0%, Recall@16 = 99.2% and Recall@32 = 99.7% respectively. It’s worth noting that the metric of Recall@1 is the most important index to analyze the effectiveness of methods. The proposed GOSL_m_ outperforms GOSL, GLSL_m_, GLSL and N-pairs with 2.9%, 3.8%, 4.3% and 3.2% respectively at Recall@1. The results of GOSL_m_ are increased by 2.9% over GOSL at Recall@1 and GLSL increases the Recall@1 by 0.5% over GOSL_m_. We could conclude that the global optimal structured loss with pairs mining scheme is superior than other DML methods and the pairs mining scheme is significant in improving the retrieval performance on the dataset of UCMD.

We could conclude according to the results in Table 9 that our proposed GOSL_m_ achieves the best performance at Recall@K (K = 1, 2, 4, 8, 16, 32) and the results are reported as Recall@1 = 94.8%, Recalll@2 = 97.0%, Recall@4 = 98.5%, Recall@8 = 99.3%, Recall@16 = 100% and Recall@32 = 100% respectively. We could find that the Recall@1 of GOSL_m_ outperforms the methods of GOSL, GLSL_m_, GLSL and N-pairs by 1.5%, 0.3%, 2.0% and 1.2% respectively. Moreover, the performance of GOSL_m_ is increased by 1.5% over GOSL and the GLSL_m_ is increased by 1.7% over GLSL at Recall@1. According to the analyses, we could know that our proposed GOSL_m_ shows great superiority and effectiveness in the task of RSIR on SATREM.

We could make a conclusion as follows from Table 10. We achieve the best results with our proposed GOSL_m_ at Recall@K (K = 1, 2, 4, 8, 16, 32) and we would show the results as Recall@1 = 97.2%, Recalll@2 = 97.5%, Recall@4 = 98.1%, Recall@8 = 98.7%, Recall@16 = 99.1% and Recall@32 = 99.5% respectively. The proposed GOSL_m_ outperforms GOSL, GLSL_m_, GLSL and N-pairs by 1.2%, 1.4%, 1.8% and 2.2% respectively at Recall@1. We observe that the methods with mining scheme could be helpful in improving the RSIR performance. To be specific, the Recall@1 of GOSL_m_ and GLSL_m_ are improved by 1.2% and 0.4% over GOSL and GLSL. We could conclude from the analyses above that our proposed global optimal structured loss with pairs mining scheme is superior than other DML methods and the pairs mining scheme is helpful in improving the retrieval performance on SIRI.

We could learn from Table 11 that the proposed GOSL_m_ obtains the best results at Recall@K (K = 1, 2, 4, 8, 16, 32) and the results are reported as Recall@1 = 91.1%, Recalll@2 = 94.3%, Recall@4 = 96.3%, Recall@8 = 97.6%, Recall@16 = 98.3% and Recall@32 = 98.7% respectively. The proposed GOSL_m_ outperforms the methods of GOSL, GLSL_m_, GLSL and N-pairs with 3.7%, 0.8%, 3.9% and 3.8% at Recall@1 respectively. We could also learn that the GLSL and our GOSL could be improved by 3.7% (from 87.4% to 91.1%) and 3.1% (from 87.2% to 90.3%) respectively at Recall@1 when utilizing the pairs mining scheme. The analyses above further demonstrate that our proposed global optimal structured loss with pairs mining scheme is more effective than other DML methods and the pairs mining scheme is significant in promoting the retrieval performance on the dataset of NWPU.

**Figure 3:**
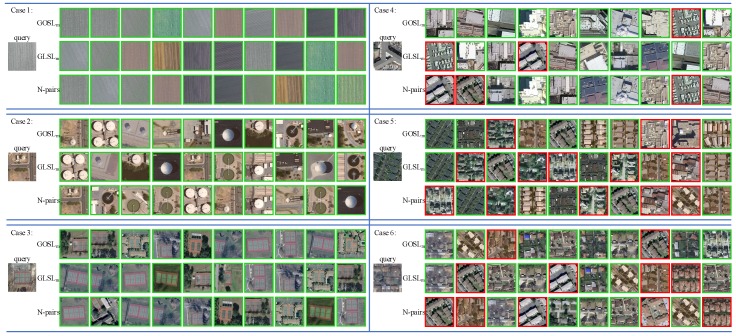
Six retrieval cases with top-10 returned results on UCMD. The left part represents three easy retrieval cases and the right part represents three hard retrieval cases. For each retrieval case, the top, middle and bottom rows denote the results obtained by using the methods of our GOSL_m_, GLSL_m_, and N-pairs. The green and red border denote true and false retrieve results respectively.

We report the errors of omission and commission with several easy and hard retrieval cases on UCMD to further validate the effectiveness of our proposed method. We show the top-10 similar images which are returned by N-pairs, GLSL_m_ and our proposed GOSL_m_ and represent the results in Figure 3. For each retrieval case, the top, middle and bottom rows denote the results obtained by using the methods of our GOSL_m_, GLSL_m_ and N-pairs. The returned images with green and red border denote true and false retrieval results respectively. We could learn from Figure 3 that there are no omission or commission on the three easy retrieve cases with the three methods which means that the three methods all achieve excellent retrieval performance for the three easy categories (i.e., agricultural, storage tanks and tennis court). However, on other three hard cases, GOSL_m_, GLSL_m_ and N-pairs perform worse as the categories of buildings, dense residential and medium residential with very low interclass variabilities. On case 4, the errors of GOSL_m_ are lower than of GLSL_m_ and N-pairs. On case 5, the errors of GOSL_m_, GLSL_m_ and N-pairs are three, five and five respectively and the results show that our proposed GOSL_m_ outperforms GLSL_m_ and N-pairs for the category of dense residential. On case 6, errors with GOSL_m_, GLSL_m_ and N-pairs are two, four and five respectively which demonstrates that our proposed GOSL_m_ is more effective than the other two DML methods. In a word, our GOSL_m_ achieves the best performance on some easy retrieval cases and exhibits great superiority in coping with the challenge of low interclass variabilities existing in most categories of remote sensing images comparing with other DML methods.

### 4.4. Ablation Study

In this section, we perform an ablation study on sensing datasets. We make analysis on hyper-parameters of our global optimal structured loss and analyze the performance of our method with different embedding size. We also study the impact of batch size for the performance of our proposed method. We would like to give more details as follows.

#### 4.4.1. Hyper-Parameter Analysis

We conduct the analysis about the main parameters which have been mentioned in Section 3 on the dataset of Google Image Dataset of SIRI-WHU [17,19,78] on the Inception network with batch normalization [75]. We set embedding size to 512 and the batch size to 40 in our experiments And we set ϵ=0.1 which is defined in Equations (11) and (12), $\beta_{1}=2$ and $\beta_{2}=50$ which are parameters in Equation (16) by following the setting of [45]. We use average value of precision (*AveP*) to measure the performance of RSIR as the same to DBOW.

The effectiveness of the fine-tuned network is crucial for more discriminative feature extraction which is significant to obtain more appreciable performance in the task of RSIR. In our proposed method, we aim to utilize a fixed positive boundary (α−m) to restrict the positive pairs into this boundary and use a given negative boundary α to force the negative pairs father than this boundary. Therefore, m is a fixed margin used to separate the two different boundaries. Herein, different values of α and m could differ the retrieval result. To achieve the best performance in RSIR task, we release our hyper-parameter analysis on α and m as follows.

As described in Section 3.4, factor α is a hyper-parameter used to limit the negative pairs far away from the positive pairs. We give a discussion on different α with {0.5,0.6,0.7,0.8,0.9,1.0} by fixing m=0.5. And we represent the results in Table 12.

We could make a conclusion from Table 12 that when α is smaller than 0.6, the *AveP* keeps increasing monotonically. On the contrary, when α is larger than 0.6, the performance would decrease. We achieve the best result 96.6% when α is 0.6. We would like to set α=0.6 in the section of experiments and discussion.

As for factor m, it is used to pull apart positive sample pairs away from negative ones. We conduct experiment to discuss the impact of hyper-parameter m by setting its value at {0.1,0.2,0.3,0.4,0.5,0.6} and fixing α to 0.6. The results are shown in Table 13.

From Table 13, we could conclude that when m is smaller than 0.5, the performance gradually increases. However, when m is larger than 0.5, the performance falls into degrading. The best result 96.6% would be achieved when m=0.5. We prefer to select m=0.5 for our following experiments according to the results in Table 13.

#### 4.4.2. Impact of Embedding Size

Referring to the work of Wang et al. [45], the embedding size during training has an important impact on the retrieval performance. We compare the effectiveness of our proposed loss function on UCMD, SATREM, SIRI and NWPU datasets with embedding size at {64, 128, 256, 512, 1024}. We set batch size as B=40. The results are reported in Table 14 and the best result is highlighted in bold. We could learn from Table 14 that the performance of UCMD, SATREM, SIRI and NWPU keeps sustained growth within the embedding size at 512 and it would go down with embedding size at 1024. The best results would be obtained when embedding size is set to 512 on the four datasets.

#### 4.4.3. Impact of Batch Size

The batch size plays an important role in DML methods as it determines the size of problems need to be processed for each iteration in the training phase. We perform a set of experiments on UCMD, SATREM, SIRI and NWPU datasets with embedding size at 512, and we set batch size to {10, 20, 40, 60, 100, 160} for comparing. We report the results in Table 15. As the number of categories is limited in each dataset, the batch size of four datasets would be limited within 100, 105, 60 and 225 respectively. Once the batch size is larger than its upper limit, the related result would be invalid. We could learn from Table 15 that batch size has different degrees of influence on the four datasets. The changes of performance remain within about 1% on UCMD and SIRI, the SATREM and NWPU is most sensitive to the variation of batch size with the performance changes from 86.5% to 91.1% and 83.9% to 90.3% respectively. We obtain the best performance on the four datasets with batch size at 40.

### 4.5. The Retrieval Execution Complexity

In this section, we analyze the retrieval execution complexity of the retrieval system with our proposed method. We measure the time (in milliseconds) required for the retrieval process which includes deep features extraction and similarity matching. During the process of deep features extraction, it takes about 10 milliseconds to extract deep features for each image with size of 224×224 which is faster than the existing fasted RSIR methods [49]. We report the results on Table 16 and compare the retrieval time (similarity matching) taken from ADLF [49].

We could learn from Table 16 that as the size of test database grows, more time would be required for retrieval and the same conclusion is reached for the embedding size. Concretely speaking, the retrieve execution time is lower than ADLF which is the existing fast methods by 1.36, 2.42, 9.64, 25.9, 45.64 and 73.63 milliseconds with DB size of 50, 100, 200, 300, 400 and 500, respectively, when the embedding size is 256. When the embedding size is 512, the retrieval execution time is lower than ADLF by 0.68, 2.97, 10.41, 15.24, 28.12 and 42.55 with DB size of 50, 100, 200, 300, 400 and 500, respectively. We achieve the lowest retrieve execution time with embedding size of 256 and the best results are 0.28, 0.40, 0.66, 1.03, 1.49 and 2.31 milliseconds at the DB size of 50, 100, 200, 300, 400 and 500, respectively. We could learn that the embedding size has less effect of lower than 2 milliseconds on the retrieval time comparing with DN7, DN8, DBOW and ADLF. Based on the discussions above, we could observe that our proposed method could achieve the state-of-the-art performance with lower retrieval time.

## 5. Conclusions

In this paper, we propose a novel global optimal structured loss under DML paradigm for more effective remote sensing image retrieval. Our proposed global optimal structured loss aims to learn an effective embedding space where the positive pairs would be limited within a given positive boundary and the negative ones would be pushed away from a fixed negative boundary, and the positive and negative pairs would be separated by a fixed margin. To deal with the key issue of local optimization in most DML methods, we propose to utilize a softmax function rather than a hinge function in our loss function to realize global optimization. To make full use of the sample pairs and take the difference and relationship between positive and negative sample pairs into consideration, we utilize a superior pairs mining strategy to mine more informative sample pairs in the confusion scope. It helps to eliminate the influence of less informative sample pairs and utilize the mined sample pairs to establish an elegant similarity structure for positive and negative sample pairs and the structure distribution could be preserved during embedding space optimization. Furthermore, our proposed global optimal structured loss would achieve the state-of-the-art performance with the lowest retrieval time on four popular remote sensing datasets compared with baselines.

Herein, we study the effectiveness of DML methods used in the task of RSIR and concentrate on how to design a more elegant loss function for more effective embedding space learning. The experimental results show that our proposed method achieves the state-of-the-art performance under the metric of *AveP* and Recall@K when compared with other common DML methods. We also improve the retrieval performance on SIRI and NWPU over the baselines by a large margin and refresh the state-of-the-art results. However, we could only achieve the second-best performance on UCMD and SATREM. It’s worth noting that we don’t conduct any post-processing operations and extra techniques like query expansion and attention mechanism on our proposed method. From the discussion we presented, our method fails to extract more informative feature representations which could be significant in improving retrieval performance. We prefer to combine the attention network with DML methods and utilize post-processing operations to further enhance the performance of RSIR in our future works.

## Figures and Tables

**Table 1:** The detail introduction of baselines.

Baseline	Feature Representations	Representation Size
DN7 [28]	Convolutional	4096
DN8 [28]	Convolutional	4096
ResNet50 [51]	Convolutional + VLAD	1500
DBOW [50]	Convolutional + BoW	16,384
ADLF [49]	Convolutional + VLAD	16,384

**Table 2:** *AveP* (%) evaluation on four different remote sensing datasets, the best results would be bolded.

Method	UCMD	SATREM	SIRI	NWPU
DN7 [28]	70.4	74.0	70.0	60.5
DN8 [28]	70.5	74.0	69.6	59.5
ResNet50 [51]	81.6	76.4	86.2	79.8
DBOW [50]	83.0	**93.3**	92.6	82.1
ADLF [49]	**91.6**	89.5	83.8	85.7
GOSLm	85.8	91.1	**96.6**	**90.3**

**Table 3:** Precision (%) of 21 geographic categories in UCMD with various RSIR methods. The best results would be highlighted in bold.

Categories	DN7 [28]	DN8 [28]	ResNet50 [51]	DBOW [50]	ADLF [49]	GOSLm
Agriculture	94	93	85	92	80	**95**
Airplane	74	75	93	95	**97**	82
Baseball	78	77	73	87	77	**90**
Beach	94	97	**99**	88	94	92
Buildings	51	47	74	**93**	85	78
Chaparral	98	98	95	94	**100**	95
Dense	36	33	62	96	90	55
Forest	98	98	87	**99**	98	95
Freeway	72	71	69	78	**99**	83
Golf	63	65	73	85	83	**92**
Harbor	85	84	97	95	**100**	95
Intersection	65	61	81	77	**86**	80
Medium-density	66	60	80	74	**92**	59
Mobile	66	65	74	76	**94**	80
Overpass	57	60	97	86	**99**	78
Parking	92	90	92	67	**99**	95
River	48	51	66	74	**87**	86
Runway	87	83	93	66	**99**	91
Sparse	67	78	69	79	79	**91**
Storage	40	45	86	50	93	**95**
Tennis	48	53	70	94	94	**95**
Average	70.4	70.5	81.6	83.0	**91.6**	85.8

**Table 4:** Precision (%) of 20 geographic categories in SATREM with various RSIR methods. The best results would be highlighted in bold.

Categories	DN7 [28]	DN8 [28]	ResNet50 [51]	DBOW [50]	ADLF [49]	GOSLm
Agriculture	85	85	86	**97**	90	92
Airplane	64	64	86	96	88	**100**
Artificial	74	78	93	97	81	**98**
Beach	68	66	86	95	87	**98**
Buildings	74	71	92	**97**	94	94
Chaparral	71	69	79	96	90	**100**
Cloud	**100**	**100**	97	99	97	**100**
Container	72	74	97	96	**100**	92
Dense	87	85	89	**100**	94	92
Factory	59	58	69	**91**	74	72
Forest	94	93	89	96	95	**98**
Harbor	60	65	80	**98**	96	**98**
Medium-density	68	66	67	**100**	67	53
Ocean	95	94	91	92	92	**100**
Parking	69	63	87	95	**96**	88
River	60	63	**83**	71	74	**83**
Road	64	60	85	82	**93**	90
Runway	84	82	96	86	**97**	**97**
Sparse	69	75	75	**92**	85	78
Storage	63	70	98	91	**100**	99
Average	74.0	74.0	86.2	**93.3**	89.5	91.1

**Table 5:** Precision (%) of 12 geographic categories in SIRI with various RSIR methods. The best results would be highlighted in bold.

Categories	DN7 [28]	DN8 [28]	ResNet50 [51]	DBOW [50]	ADLF [49]	GOSLm
Agriculture	82	79	95	99	94	**100**
Commercial	80	80	90	99	97	**100**
Harbor	55	56	63	89	74	**98**
Idle	58	60	63	97	80	**99**
Industrial	72	70	88	90	96	**98**
Meadow	71	63	77	93	82	**95**
Overpass	71	76	80	89	94	**100**
Park	67	67	82	87	90	**100**
Pond	47	50	57	**97**	74	96
Residential	81	78	84	97	94	**98**
River	59	57	44	**89**	69	77
Water	99	99	94	86	99	**100**
Average	69.9	69.5	76.4	92.6	86.9	96.6

**Table 6:** Precision (%) of 45 geographic categories in NWPU with various RSIR methods. The best results would be highlighted in bold.

Categories	DN7 [28]	DN8 [28]	ResNet50 [51]	DBOW [50]	ADLF [49]	GOSLm
Airplane	56	57	88	**98**	93	96
Airport	50	47	72	**95**	81	90
Baseball Diamond	43	45	69	86	64	**93**
Basketball Court	33	32	61	83	71	**90**
Beach	56	58	77	85	83	**96**
Bridge	67	66	73	**95**	81	93
Chaparral	93	93	98	96	**99**	98
Church	25	26	56	**80**	64	64
Circular Farmland	83	84	97	94	**99**	97
Cloud	91	91	92	**98**	**98**	**98**
Commercial Area	53	45	82	79	**88**	78
Dense Residential	62	58	89	90	**95**	92
Desert	85	83	87	**97**	92	90
Forest	91	89	95	95	**97**	94
Freeway	55	52	65	64	86	**88**
Golf Course	63	60	96	82	**97**	96
Ground Track Field	59	61	63	80	77	**96**
Harbor	64	65	93	88	97	**99**
Industrial Area	57	52	75	85	88	**90**
Intersection	57	51	64	80	72	**97**
Island	78	73	88	88	**94**	93
Lake	69	69	80	85	85	**89**
Meadow	82	82	84	90	**93**	**93**
Medium Residential	57	51	78	**94**	77	82
Mobile Home Park	52	52	93	83	**97**	94
Mountain	74	71	88	95	**96**	86
Overpass	51	53	87	74	90	**95**
Palace	25	23	41	**80**	56	51
Parking Lot	71	68	95	70	97	**98**
Railway	60	58	88	84	**89**	77
Railway Station	48	46	62	86	73	81
Rectangular Farmland	71	66	82	66	**88**	86
River	50	50	70	76	75	**90**
Roundabout	61	61	72	83	90	**95**
Runway	63	58	80	78	89	**90**
Sea Ice	91	89	98	90	**99**	**99**
Ship	43	46	61	65	69	**95**
Snowberg	78	79	97	83	98	**99**
Sparse Residential	58	62	69	84	70	**93**
Stadium	59	57	81	57	86	**92**
Storage Tank	61	62	88	48	94	**98**
Tennis Court	34	37	80	72	78	**95**
Terrace	54	54	88	76	**90**	89
Thermal Power Station	43	45	68	72	78	**89**
Wetland	50	49	82	70	80	**85**
Average	60.5	59.4	79.8	82.1	85.7	**90.3**

**Table 7:** *AveP* (%) evaluated on four different remote sensing datasets. The best results would be bold.

Method	UCMD	SATREM	SIRI	NWPU
N-pairs	82.2	85.3	92.8	84.3
GLSL	82.6	85.1	94.9	85.5
GLSL_m_	84.3	87.2	95.2	88.6
GOSL	85.1	86.8	95.3	85.8
GOSL_m_	**85.8**	**91.1**	**96.6**	**90.3**

**Table 8:** Recall@K (%) evaluated on UCMD. The best results would be bold.

Recall@K (%)	1	2	4	8	16	32
N-pairs	95.3	98.3	98.5	**99.0**	**99.2**	**99.7**
GLSL	94.2	96.1	96.9	98.3	98.3	99.5
GLSL_m_	94.7	96.4	97.1	97.6	98.1	**99.7**
GOSL	95.4	98.1	98.3	98.5	99.0	**99.7**
GOSL_m_	**98.5**	**98.8**	**99.0**	**99.0**	**99.2**	**99.7**

**Table 9:** Recall@K (%) evaluated on SATREM. The best results would be bold.

Recall@K (%)	1	2	4	8	16	32
**N-pairs**	93.6	95.6	97.5	98.6	99.3	99.8
**GLSL**	92.8	96.5	97.3	98.3	99.3	99.6
**GLSL_m_**	94.5	97.1	98.6	99.5	99.6	99.6
**GOSL**	93.3	96.0	98.0	98.5	99.3	99.6
**GOSL_m_**	**94.8**	**97.0**	**98.5**	**99.3**	**100**	**100**

**Table 10:** Recall@K (%) evaluated on SIRI. The best results would be bold.

Recall@K (%)	1	2	4	8	16	32
N-pairs	95.0	96.0	96.8	97.7	98.5	99.5
GLSL	95.4	96.2	97.5	98.1	98.9	98.9
GLSL_m_	95.8	96.4	96.8	98.1	98.5	99.5
GOSL	96.0	96.6	97.2	97.5	97.9	98.7
GOSL_m_	**97.2**	**97.5**	**98.1**	**98.7**	**99.1**	**99.5**

**Table 11:** Recall@K (%) evaluated on NWPU. The best results would be bold.

Recall@K (%)	1	2	4	8	16	32
N-pairs	87.3	92.5	95.1	96.9	98.0	**98.7**
GLSL	87.2	91.0	93.0	94.5	95.3	96.0
GLSL_m_	90.3	93.6	95.8	97.1	98.0	98.5
GOSL	87.4	91.2	93.3	94.8	95.7	96.1
GOSL_m_	**91.1**	**94.3**	**96.3**	**97.6**	**98.3**	**98.7**

**Table 12:** The *AveP* (%) on different α with {0.5,0.6,0.7,0.8,0.9,1.0} on SIRI-WHU with m=0.5. The best results would be highlighted in bold.

*α*	0.5	0.6	0.7	0.8	0.9	1
*AveP* (%)	96.3	**96.6**	96.1	96.0	95.8	95.7

**Table 13:** The *AveP* (%) on different m with {0.1,0.2,0.3,0.4,0.5,0.6} on SIRI-WHU with α=0.6. The best results would be bold.

*m*	0.1	0.2	0.3	0.4	0.5	0.6
*AveP* (%)	95.4	95.6	95.6	95.8	**96.6**	96.0

**Table 14:** *AveP* (%) comparison on our proposed method with embedding size at {64, 128, 256, 512, 1024}. The best results would be highlighted in bold.

*AveP* (%)	64	128	256	512	1024
UCMD	84.4	85.0	85.1	**85.8**	85.6
SATREM	85.2	85.6	86.8	**91.1**	86.9
SIRI	95.2	95.9	96.0	**96.6**	95.9
NWPU	87.9	88.2	88.6	**90.3**	88.8

**Table 15:** *AveP* (%) comparison on our proposed method with batch size at {10, 20, 40, 60, 100, 160}. The “-” denotes the related results are invalid. The best results would be bold.

*AveP* (%)	10	20	40	60	100	160
UCMD	84.7	85.7	**85.8**	85.6	85.5	-
SATREM	86.5	88.3	**91.1**	86.5	86.1	-
SIRI	95.5	95.6	**96.6**	95.5	-	-
NWPU	83.9	87.3	**90.3**	88.1	88.4	85.9

**Table 16:** Retrieval time (in milliseconds) with various test datasets and embedding size. The best results would be in bold.

DB Size	DN7 [50]	DN8 [50]	DBOW [50]	ADLF (1024) [49]	ADLF (512) [49]	ADLF (256) [49]	GOSLm (1024)	GOSLm (512)	GOSLm (256)
50	5.80	5.70	2.30	1.70	0.97	0.61	0.34	0.29	**0.28**
100	17.10	17.30	6.10	3.31	3.43	1.85	0.89	0.46	**0.40**
200	58.70	58.40	21.40	11.54	11.13	6.43	1.90	0.72	**0.66**
300	127.40	127.80	45.90	28.18	16.56	10.72	2.59	1.32	**1.03**
400	223.10	224.30	79.60	49.01	29.72	14.87	3.37	1.60	**1.49**
500	246.00	344.90	123.90	77.83	44.90	22.98	4.20	2.35	**2.31**
